# Safety study of leucoreduced allogeneic pooled freeze-dried platelet-rich plasma in healthy equine joints

**DOI:** 10.3389/fvets.2025.1625431

**Published:** 2025-07-14

**Authors:** Sarah Kooy, Jemma Constant, Robert Cole, Lindsey Boone

**Affiliations:** ^1^Department of Clinical Sciences, College of Veterinary Medicine, Auburn University, Auburn, AL, United States; ^2^College of Veterinary Medicine, Auburn University, Auburn, AL, United States

**Keywords:** equine, platelet-rich plasma, pooled, allogeneic, freeze-dried

## Abstract

**Introduction:**

Clinical use of blood-derived intra-articular therapies, such as platelet-rich plasma (PRP), have increased in equine athletes due to their proposed disease-modifying effects. Need for a shelf-stable, allogeneic PRP product with known composition for standardized treatment exists. The objective of this study was to compare systemic and local effects of a single intra-articular injection of equine leucoreduced allogeneic pooled freeze-dried PRP (alloPRP) to a placebo control (saline) in normal, healthy equine joints.

**Methods:**

Twelve healthy horses were randomly assigned to either control (saline) or treatment (alloPRP) (*n* = 6 horses per group). The study used a blinded 2-period, 2-treatment, 2-joint non-crossover experimental design. Each study period was defined as 1 week, followed by a 2-week washout between study periods. One joint (radiocarpal or tarsocrural joint) was treated and evaluated per study period. Treatment sequence and limbs chosen for treatment were randomized for each horse. Systemic effects were measured at different time points by physical examination, bloodwork (complete blood count and serum biochemistry), and lameness examination (subjective and objective). Local effects to the joint measured at different time points included heat, swelling, joint circumference, and response to passive flexion as well as synovial fluid analysis. Data was analyzed using linear mixed models with significance set at *p* < 0.05.

**Results:**

There were no differences between groups for joint swelling (*p* = 0.40), joint circumference (*p* = 0.55), heat scores (*p* = 0.09), or passive flexion (*p =* 0.70) following intra-articular injections. Both subjective and objective lameness scores were no different on any study day for both treatment (*p =* 0.47) and control groups (*p =* 0.31). There were no differences in complete blood count or serum biochemistry values between groups. No difference in synovial fluid TNCC (*p* = 0.80), TP (*p* = 0.94), or synovial fluid concentrations of TNFα (*p =* 0.45) and IL-1raP (*p =* 0.18) were observed between groups for each sampled time point (T0 and T7d).

**Conclusion:**

The alloPRP formulation was demonstrated to be safe for use in equine joints. Further investigation is necessary for evaluation of clinical efficacy.

## 1 Introduction

Osteoarthritis (OA) is the most common cause of chronic lameness in the horse, with increasing prevalence due to age and sport-related performance (1, 2). Conventional therapies for OA, i.e., corticosteroids, focus on reducing the symptoms of disease with limited evidence for disease modification (3, 4). Additionally, intra-articular administration of corticosteroids can be harmful to articular tissues due to frequent, repeat administration and dosage (5). Therapies that can both modify inflammation and promote tissue repair are needed.

Blood-derived orthobiologic products, such as platelet-rich plasma (PRP), have been developed to harness important anti-inflammatory and anabolic proteins as well as other bioactive molecules to help modulate the complex inflammatory pathways of the synovium and articular cartilage to promote tissue repair (3). PRP is a plasma product with a greater number of platelets than whole blood. Platelets contain alpha granules which harbor numerous growth factors, cytokines and mitogens important for wound repair, however, the process of concentrating platelets brings along other blood components, such as white and red blood cells. The concentration of the white blood cell (leukocyte) component and the effects of leukocytes on tissue healing remains controversial. Current research suggests that while leukocyte reduction of PRP may be beneficial, it is the phenotypic characterization of concentrated leukocytes that may be most important for tissue repair. Regardless, PRP has been shown *in vitro* to have disease modifying effects on cartilage repair (6, 7). PRP has also been shown to improve patient reported outcomes in humans with knee OA (8) and clinical benefits of PRP for joint-related lameness have been reported in the horse (9).

Our understanding of the clinical effects of platelet-derived biologics is marred by inability for study comparison due to product variability due to different preparation methods as well as inconsistency in product nomenclature and standards for product characterization. There are also both patient and treatment-related factors that make it challenging to fully understand the clinical benefits. Pooling blood-derived products from multiple, healthy donors capitalizes on the natural variability that exists between patients related to cellular composition as well as concentration and inclusion of various bioactive molecules including growth factors and cytokines, producing a more homogenous product (10, 11). Furthermore, preservation and storage of platelets using lyophilization or freeze drying to allow long-term storage in ready-to-use, shelf-stable products has been evaluated to enhance accessibility for treatment. Maintenance of protein integrity and activity following lyophilization of equine and human platelet derived products has been demonstrated (12–14). These factors have contributed to the need for and development of convenient and consistent biologic products for use in equine musculoskeletal repair.

Pooling of platelet derived products from various donors delivers an allogeneic product that could incite an immune response from the recipient. Despite these concerns, allogeneic PRP has been shown to be safe and efficacious for treatment of musculoskeletal disease in both human and animals (15–17). Safety of autologous frozen and allogeneic freeze-dried PRP administered intra-articularly to healthy equine joints has been reported. Both autologous frozen and allogeneic freeze-dried PRP treatments induced a mild, self-limiting inflammatory reaction. No differences in systemic or local responses were observed between autologous frozen and allogeneic freeze-dried PRP leading to the conclusion that allogeneic freeze-dried PRP is safe for use in horses (11).

More recently, a leucoreduced allogeneic pooled freeze-dried PRP product (PrecisePRP™) has been made which contains partially activated platelets stored at room temperature and rehydrated with sterile water for clinical use. The objective of this study was to compare the safety of intra-articular injection of this leucoreduced allogeneic pooled freeze-dried equine PRP product (PrecisePRP™) to a placebo control (saline) in a masked, randomized experiment. We hypothesized that intra-articular injection of the alloPRP product causes similar systemic and local changes after injection as a placebo control.

## 2 Materials and methods

### 2.1 Study design

**Figure 1:**
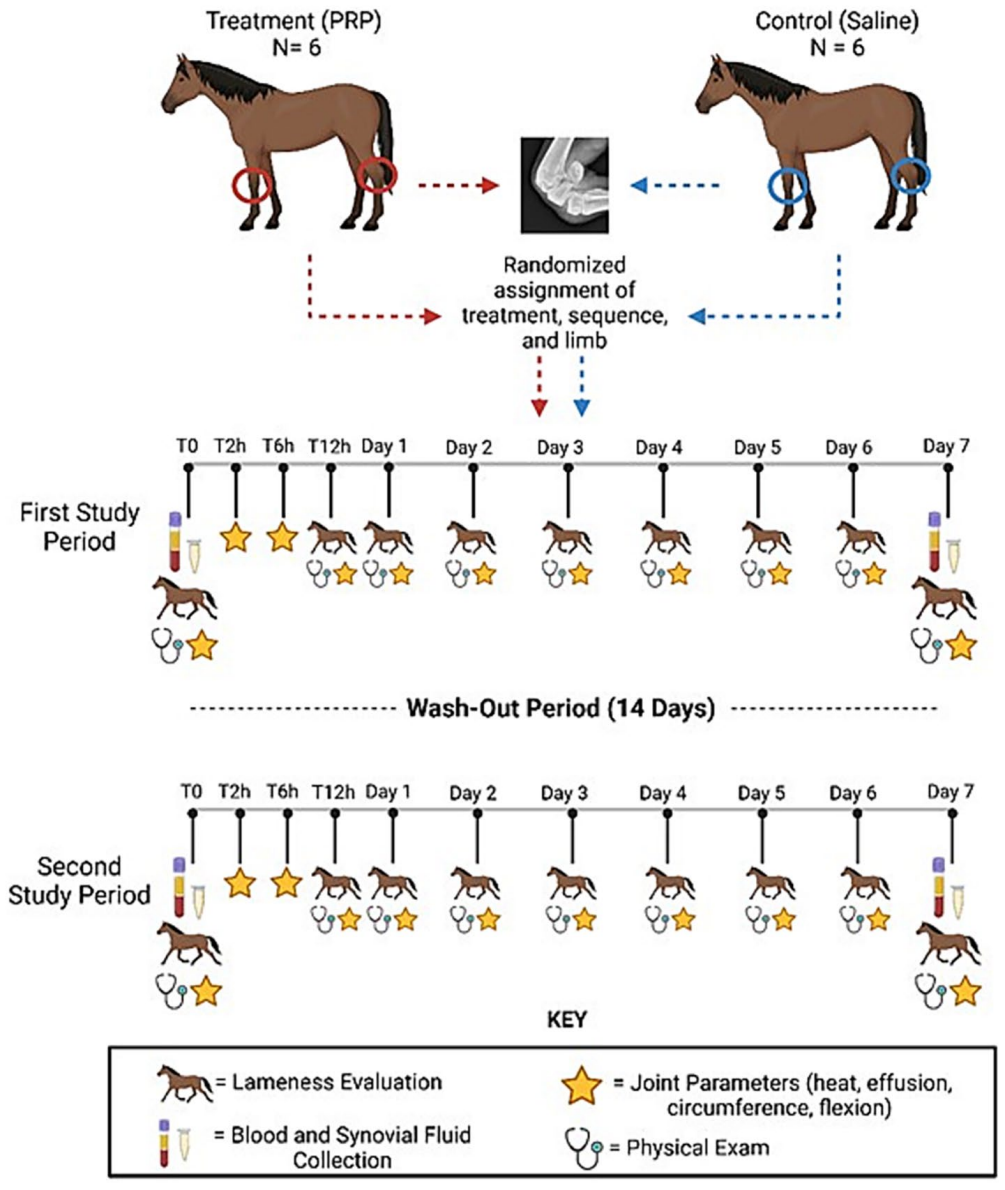
Study design. Blinded 2-period, 2-treatment, 2-joint non-crossover experimental design. Horses were treated with either pooled leucoreduced allogeneic freeze-dried PRP or saline control. This figure was created using Biorender.com.

The study used a blinded 2-period, 2-treatment, 2 joint non-crossover experimental design (Figure 1). Each study period was defined as 1 week, followed by a 2-week washout between study periods. Each horse was randomly assigned to either control (0.9% sodium chloride) or treatment (alloPRP) group. For each horse, one radiocarpal joint (RCJ) and one tarsocrural joint (TCJ) were randomly assigned to treatment. One joint was treated and evaluated per study period. Treatment sequence and limbs chosen for treatment were randomized for each horse. The individual preparing and performing the joint treatments (SK) was not the same individual performing the evaluations post-treatment to maintain blinding (LB).

The study protocol was reviewed and approved by the Auburn University Institutional Animal Care and Use committee (IACUC # 2023-5204). Twelve horses (4 Thoroughbreds, 5 Quarter Horses, 2 Draft crosses, and 1 Warmblood) were selected from the university research heard for study enrollment. The ages of horses ranged from 8 to 18 years (mean, 12.5 years). Horses were assessed for suitability of study inclusion. Assessment included a complete physical and musculoskeletal examination. Included horses were not free of baseline lameness due to the nature of horses obtained and maintained within the University research herd. Horses were included in the study if there were no radiographic abnormalities of the radiocarpal and tarsocrural joints as evaluated by a board-certified veterinary radiologist (RC). A complete blood count (CBC) and chemistry panel were performed for each horse at the beginning of the study to rule out systemic disease.

The product used, PrecisePRP™ Equine (VetStem, Poway, CA), contains a leucoreduced, allogeneic, pooled platelet-rich plasma as the active agent rehydrated with 8 ml of sterile saline. The intended clinical dose from the manufacturer is 4 ml per joint and was the dose used for this study. Platelet rich plasma was obtained via apheresis and pooled from 18 healthy donors that are part of a USDA licensed herd. The final product contained an average of 400,000 platelets/μl and 1,500 white blood cells/μl. The final product also contained an average of 804 pg/ml of platelet derived growth factor (PDFG-BB). The product was tested for quality control including testing for platelet surface markers (cluster of differentiation 9 and 61), sterility, mycoplasma and endotoxin. Product used for the study was from the same lot of manufacturing (Lot # QB1617C).

### 2.2 Synovial fluid collection and joint treatments

Each horse was sedated with xylazine hydrochloride (0.4–0.5 mg/kg) given intravenously. The randomly assigned RCJ or TCJ was treated with either 4 ml of 0.9% sodium chloride or alloPRP for each study period according to sequence randomization. The joint assigned was aseptically prepared with alternating application of chlorhexidine scrub and 70% isopropyl alcohol for 5 min. The TCJ was sampled and injected from the dorsomedial approach. The RCJ was sampled and injected from the traditional dorsolateral approach with the carpus maintained in flexion during synoviocentesis. Synovial fluid (3 ml) was obtained immediately prior to administration of assigned treatment. Samples were transferred to a collection tube containing EDTA and stored at −80°C until analyzed.

If the horse was assigned to receive the alloPRP, it was prepared in a sterile manner. A vented clave was applied to the top of the vial containing the freeze-dried PRP product. The vial was held in a tilted fashion and a total of 8 ml of sterile water, per the company reconstitution directions, was administered slowly through the clave hub, flowing down the side of the vial for rehydration. The fluid vial was gently swirled to avoid excess foaming while reconstituting the product. Once all of the powder appeared dissolved, 4 ml of reconstituted alloPRP was drawn into a sterile syringe through the clave for injection into the assigned joint.

### 2.3 Monitoring of physical examination parameters

Horses were individually housed in a stall and monitored daily during the sampling period and for an additional 12-h following collection of the post-treatment synovial fluid samples. Horses were monitored throughout the study period for changes in temperature, pulse, and respiration. Parameters were monitored just prior to treatment (T0), every 12 h for 96 h and then every 24 h for 7 days to conclude the study period.

The RCJ and TCJ were subjectively scored by digital palpation for heat, joint effusion, and response to flexion at T0h, T2h T6h, T12h, T24h and then every 24 h until the end of the study period. Heat was graded by palpation of the treated joint and graded from 0 to 3 (0 = none, 1 = minor, 2 = moderate, and 3 = severe). Joint swelling was graded from 0 to 4 (0 = no swelling; 1 = minimal swelling localized to the injection site; 2 = mild swelling localized to the RCJ/TCJ; 3 = moderate swelling extending to encompass the carpus or tarsus; and 4 = marked swelling extending above or below the carpus or tarsus). Joint circumference (mm) was measured at the midline of the injected joint using a standard cloth measuring tape, with the location of measurement marked medial and laterally with horizontal clip markings to maintain consistency.

Response to passive flexion was subjectively graded from 0 to 3 (0 = none; 1 = minor; 2 = moderate; 3 = severe). Lameness evaluations were performed on each horse at T0, T12h and then every 24 h until the end of the study period Objective lameness assessments were performed with a body-mounted intertial sensor system (Equinosis Q with Lameness Locator®, Columbia, MO). Horses were trotted on a straight line by a handler on the same firm surface for a minimum of 25 strides.

On day 7 (T168h) post-treatment, horses were sedated with xylazine hydrochloride (0.2–0.5 mg/kg IV) following their lameness evaluation. The previously treated joints were aseptically prepared. Approximately 3–5 ml of synovial fluid was collected in a sterile manner. Samples were then transferred to a collection tube containing EDTA and stored at −80°C until analyses.

### 2.4 Measured clinicopathologic parameters

For the complete blood count and serum blood biochemistry, blood was obtained from the left or right jugular vein and submitted to an outside laboratory (Idexx BioAnalytics, West Sacramento, CA) for complete blood count and biochemistry profile on T1d, T7d, T14d, and T21d by board-certified pathologists.

For synovial fluid analysis, approximately 1 ml of synovial fluid was aliquoted from the parent sample and shipped overnight on ice for analysis using the same outside laboratory as for the CBC and chemistry. Measured parameters included total nucleated cell count (TNCC), total red blood cells (RBC), and total protein (TP) (Siemens Healthcare Diagnostics, Erlangen, Germany). Differential cell counts were performed by board-certified veterinary clinical pathologists. The remaining synovial fluid was aliquoted into Eppendorf tubes, centrifuged (600 RPM for 10 min at 4°C), supernatant removed and stored at −80°C until further analysis.

For synovial fluid cytokine analysis, thawed synovial fluid samples (200 μl) were hyaluronidase-digested (10 μl of 100 IU hyaluronidase/ml acetate buffer; Worthington Biochemical Corporation, Lakewood, NJ) for 30 min at 37°C, centrifuged (12,000 RPM for 10 min; 4°C), and supernatant recovered. TNFα and IL-1 receptor antagonist protein (IL-1raP) were quantified by ELISA (R&D Systems, Minneapolis, MN).

### 2.5 Data analyses

All analyses were performed using SAS 9.4 (Cary, NC). A significance level of 0.05 was used. The assumption of normality was evaluated via inspection of QQ- and PP-plots, histograms, and skewness. Normally distributed variables were summarized descriptively with mean and standard deviation (SD). Non-normally distributed variables were summarized descriptively with median and interquartile range (IQR).

Linear mixed models (LMM) were used to test effects of treatment on temperature, CBC and chemical parameters, and synovial fluid RBC. Negative binomial generalized linear mixed models (GLMM) with a negative binomial distribution and a log link function were used to test for effects of treatment on pulse and respiratory rates. Ordinal logistic GLMMs with a multinomial distribution and a cumulative logit link function were used to test effects of treatment on joint effusion, heat, and lameness scores. Logistic GLMM with a binomial distribution and a logit link function were used to test for effects of treatment on passive flexion. These models had a fixed factor for treatment, a study day covariate and a study day by treatment interaction factor and a random intercept for each horse and for each limb within each horse. LMM assumptions (normality and homoscedasticity of model residuals) were evaluated via inspection of conditional QQ-plots, histograms, and residual plots. Satterthwaite degrees of freedom method were used in all models. REML estimation for LMM and residual pseudo-likelihood for GLMMs were used. *p*-values from the study day by treatment interaction effect were reported to test for differences in change in endpoint over time between treatment and control.

Vector sum (VS) data obtained via Lameness Locator® were not-normally distributed so Mann–Whitney tests were used to compare VS measurements between treatments on each study day for treated or untreated limbs separately.

## 3 Results

### 3.1 Physical examination findings

All horses, except for one in the control group (Horse 1), maintained heart rates, respiratory rates, and rectal temperatures within normal limits throughout the study period. There were no differences in change over time between treatment and control groups for heart rate (*p* = 0.33), respiration rate (*p* = 0.60), or temperature (*p* = 0.90). Horse 1 experienced an acute episode of colic, presumptively due to an ileal impaction that was treated medically. This resulted in a temporary elevation in heart rate (60 bpm), that resolved within 6 h following medical management.

There were no differences between groups in the change of joint effusion score up to and including day 1 (*p* = 0.40) and days 2–7 (*p* = 0.73). For joint circumference, there was no difference between groups in the change in joint circumference from day 0 to day 7 (*p* = 0.55). There was no difference between treatments in change of heat scores up to and including day 1 or days 2–7 (*p* = 0.09). There was no difference in passive flexion score between groups (*p* = 0.70). For joint evaluations, there was a trend for higher scores at 6–12 h post-injection with no significant differences present between groups. The control group displayed greater joint swelling in the first 6–12 h after first injection whereas the treatment group experienced joint swelling 6–12 h after the second injection which resolved over time.

**Figure 2:**
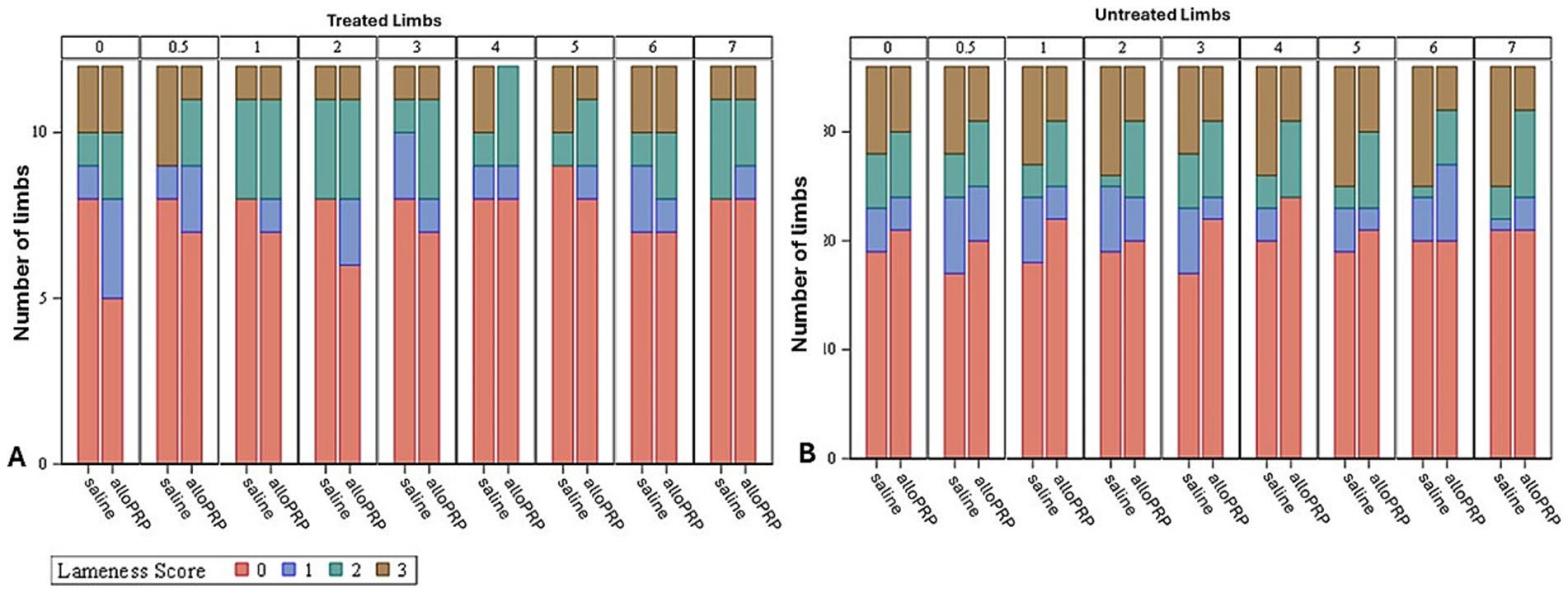
Subjective lameness scores of treated **(A)** and untreated **(B)** limbs for each study day and treatment. Study day is depicted numerically at the top of each column.

**Figure 3:**
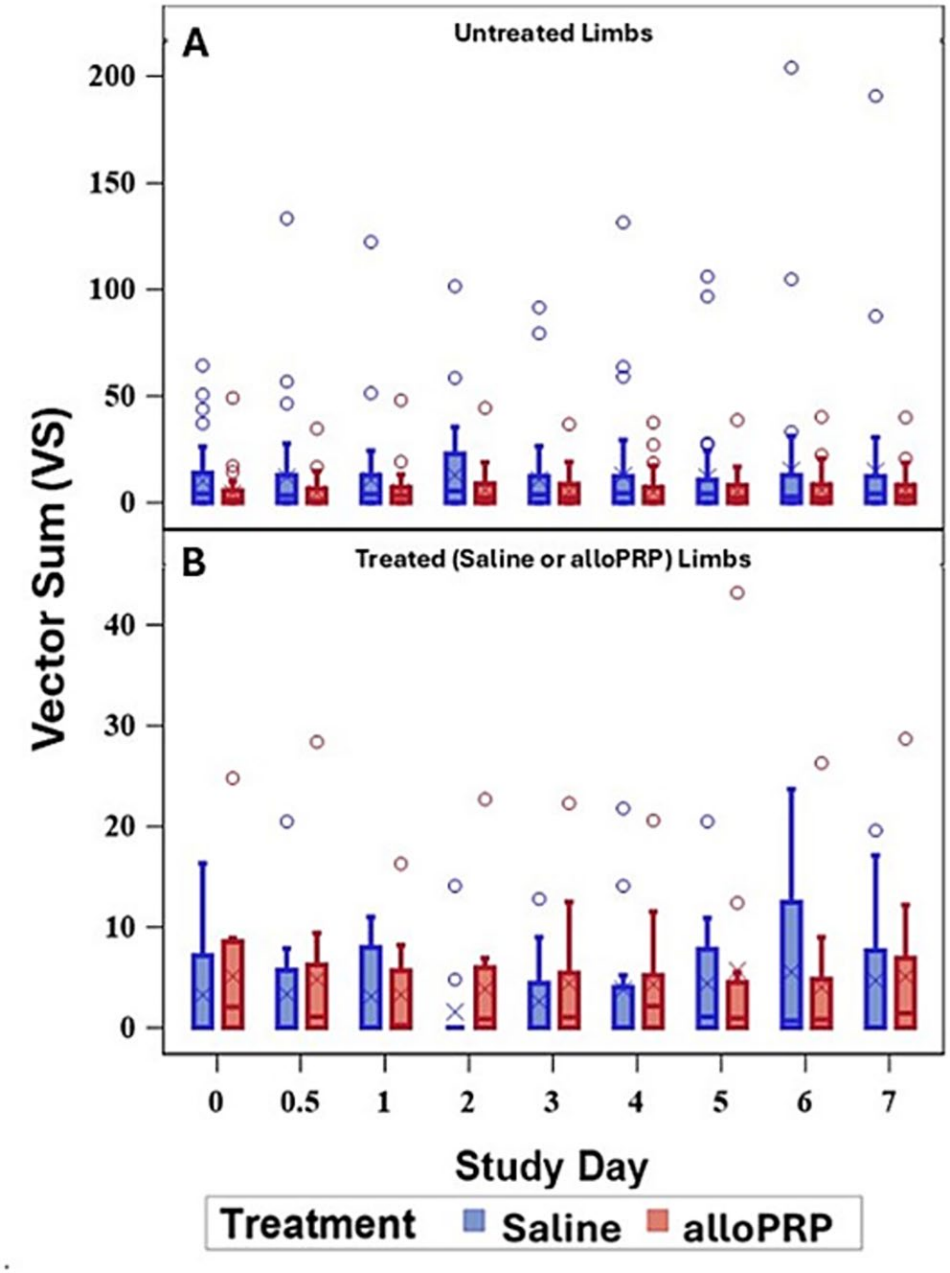
VS for treated **(A)** and untreated **(B)** limbs by study day for each treatment (saline or alloPRP). Each box is drawn from the 25th percentile to the 75th percentile. The horizontal line inside the box shows the location of the median and the symbol shows the location of the mean. Whiskers extend from the upper edge of the box to the largest observed value less than or equal to 1.5x IQR above the 75th percentile, and from the lower edge of the box to the smallest observed value greater than or equal to 1.5x IQR below the 25th percentile. Observations outside the whiskers are identified with circular points.

**Figure 4:**
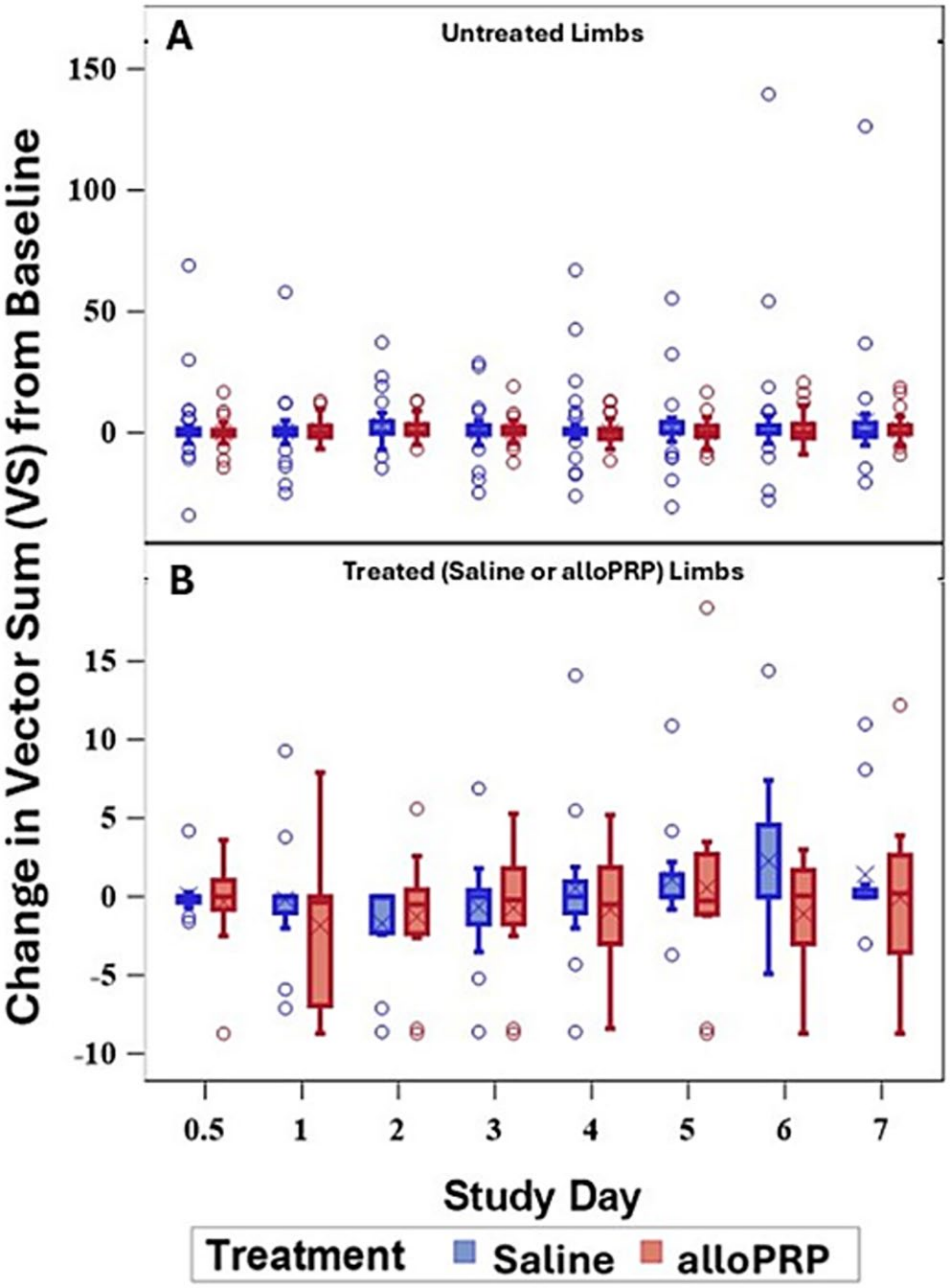
VS change from baseline of untreated **(A)** and treated **(B)** by study day and for each treatment (saline or alloPRP). Each box is drawn from the 25th percentile to the 75th percentile. The horizontal line inside the box shows the location of the median and the symbol shows the location of the mean. Whiskers extend from the upper edge of the box to the largest observed value less than or equal to 1.5x IQR above the 75th percentile, and from the lower edge of the box to the smallest observed value greater than or equal to 1.5x IQR below the 25th percentile. Observations outside the whiskers are identified with circular points.

**Figure 5:**
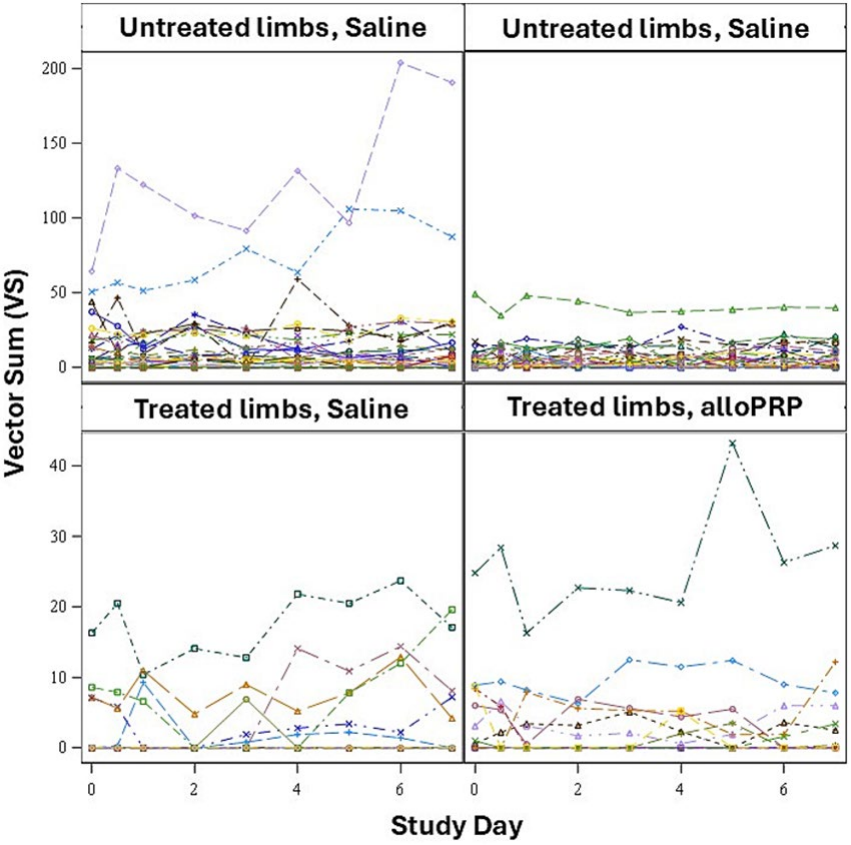
VS for each limb (*n* = 24 treated, *n* = 72 untreated) for each study day for treated and untreated limbs separately.

Baseline lameness evaluations revealed that 2/12 (16.7%) horses displayed a right forelimb lameness, 7/12 (58.3%) displayed a left forelimb lameness, 7/12 (58.3%) horses displayed a right hind limb lameness, and 5/12 (41.7%) horses displayed a left hind limb lameness. A total of 9/12 (75%) horses were lame in both a forelimb and a hind limb on baseline evaluations (3 were LF/LH, 4 were LF/RH, 1 was RF/LH, and 1 was RF/RH). The mean lameness grade for all horses was a 2.14 out of 5 following the AAEP Lameness Grading Scale. There were no differences in subjective lameness scores on any study day in treated (*p* = 0.47) or control (*p* = 0.31) limbs (Figures 2A,B). There were no differences in VS (Figure 3) or change in VS from baseline (Figure 4) between treatment groups for treated and untreated limbs on any study day for either group. The lameness data is charted for each individual horse through the study period in Figure 5.

### 3.2 Blood clinicopathologic findings

No clinically significant abnormalities were present on the CBC and chemistry profiles for all horses prior to treatment. When comparing CBC data, there were no significant changes in total monocyte count (*p* = 0.24), total neutrophil count (*p* = 0.06), neutrophil percentage (*p* = 0.67), or total WBC count (*p* = 0.05) between day T0 and T7d between groups. There was a significant change in monocyte percentage from day T0 to day T7d between groups where the saline group had a greater decline in monocyte percentage from day T0 to day T7d compared to the treatment group (*p* = 0.004). There were no differences for changes in total monocyte count (*p* = 0.12), monocyte percentage (*p* = 0.28), total neutrophil count (*p* = 0.27), neutrophil percentage (*p* = 0.957), and total WBC count (*p* = 0.17) from T0 to T7d between groups. When evaluating the blood chemistry, there were no differences observed in the change from day T0 to day T7d between groups for blood glucose (*p* = 0.34), blood urea nitrogen (*p* = 0.5), creatinine (*p* = 0.32), total protein (*p* = 0.38), alkaline phosphatase (*p* = 0.49), alanine transaminase (*p* = 0.47), and creatinine kinase (*p* = 0.82). No differences between T0h and T7d within groups observed for blood glucose (*p* = 0.62), blood urea nitrogen (*p* = 0.43), creatinine (*p* = 0.41), total protein (*p* = 0.85), alkaline phosphatase (*p* = 0.44), alanine transaminase (*p* = 0.55), and creatinine kinase (*p* = 0.13).

### 3.3 Synovial fluid clinicopathologic findings

No differences in synovial fluid clarity and color between T0 and T7d were observed. Samples were predominantly straw-colored and clear to mildly hazy throughout the study period. Analyses of synovial fluid revealed no differences in RBC concentration between time points (*p* = 0.82) or between groups (*p* = 0.80). No differences in TNCC were present between time points (*p* = 0.16) or between groups (*p* = 0.80). Similarly, no differences in total TP were present between groups (*p* = 0.94) or time points (*p* = 0.29).

No differences in synovial fluid concentrations of TNFα were observed between groups (*p* = 0.45) or within groups between T0h and T7d (*p* = 0.32). Similarly, no differences in synovial fluid concentrations of IL-1raP were observed between groups (*p* = 0.18) and within groups between T0h and T7d (*p* = 0.61).

## 4 Discussion

The main goal of this study was to perform a safety evaluation of a pooled leucoreduced, allogeneic PRP product in healthy equine joints by assessing changes to physical exam, lameness exam, and synovial fluid parameters. During the study, no adverse reactions associated with intra-articular treatment of alloPRP occurred with no significant differences in systemic or local effects between control and treatment groups. Intra-articular treatment resulted in a transitory, inflammatory response with observed increases in heat and joint swelling, but no change in lameness. This inflammatory response was similar for control and treatment joints; however, the response was more readily observed after the second alloPRP injection. The observed acute, self-limiting inflammatory response observed in both groups is likely due to the arthrocentesis itself and is similar those observed following biologic intra-articular therapies in previous studies (11, 18–20).

While both treatments elicited similar systemic responses, it is important to note that one horse did experience an acute episode of colic, likely due to the transition from pasture to stall during the study period. This colic episode was treated medically, with administration of two doses of a non-steroidal anti-inflammatory medication (flunixin meglumine, 1.1 mg/kg IV q12 h), administration of an oral electrolyte solution once via nasogastric intubation and withholding food for 12 h. This horse was in the control group. While non-steroidal anti-inflammatory drug administration could affect systemic and local inflammatory response, this mild transitory episode of colic requiring rescue analgesia did not result in outliers to the data, except for a transitory increase in heart rate. Therefore, this horse was removed from the heart rate analyses.

It is important to note that this study did not use a cross-over design with horses receiving two treatments of the same product administered into different joints. As previously discussed, PRP is considered a cellular product, therefore, both a cellular and humoral response leading to immune rejection is possible. This is important to consider with pooling from multiple donors and with consideration that most practitioners are using repeat dosing protocols when using PRP for musculoskeletal repair (21). Allo-antibodies have been demonstrated after allogeneic administration of equine mesenchymal stem cells (22) which have historically been considered immune privileged. PRP is a cellular product, but the cellular components are not immune privileged which could lead to a heightened inflammatory response with repeat dosing and/or immune rejection. Pooling from multiple donors may also be of concern, however, Colbath et al. showed no differences in synovial response following injection of autologous bone marrow derived mesenchymal stem cells compared to pooled allogeneic bone marrow derived mesenchymal stem cells, indicating that the immune response from pooled allogeneic cells may be similar to allogeneic cells form a single donor (23). To the author’s knowledge, there are no studies that have evaluated the humoral response to allogeneic PRP, pooled or not pooled, in the horse. While better understanding of immune response to allogeneic pooled PRP are needed, this study demonstrates safety like the study of Garbin et al. (11).

Certain equine joints have been shown to differ in their systemic and local response following an inflammatory event (24). Marked differences in the measured local response of the RC and TCJ were not observed, but this could be due to the degree of incited inflammation, with saline and alloPRP producing a limited inflammatory synovial response compared to IL-1ß injection.

Synovial fluid analyses demonstrated no significant differences in TP or TNCC between treatment groups as well as cytokines, TNFα and IL-1rap. Previous studies have shown peak inflammatory response with the synovial compartment to occur within 24 and up to 48 h postinjection (11). For example, PGE_2_ has been shown to reach peak levels between 3 and 6 h after intra-articular injection, being only statistically increased up to 48 h after treatment (19, 25). The injection of a hemoderived product has previously been shown to increase nucleated cell count at 6 and 24 h after treatment (11, 18). The degree of response is multifactorial, related to cellular damage caused by synoviocentesis, the physiologic and/or pathologic state of the synovial environment as well as the evoked inflammatory and immune response due to the injected material. In this study horses did not show significant changes to objective and subjective lameness, heat scores, joint swelling, or response to passive flexion at T6h and T12h. Therefore, we are left to deduce that the inflammatory reaction in the treated joints was mild in each group based on these measured parameters at post-injection times when inflammatory mediators are known to be at their peak within the joint.

This study had several limitations that are important to discuss. First, is the modest sample size with a non-crossover study design that could have resulted in the study being underpowered and therefore, true differences or subtle adverse effects may not have been captured. As previously mentioned, sampling of synovial fluid within the first 48-h post-injection would have allowed a better understanding of the incited cellular immune response to injection of alloPRP compared to saline. Repeating synoviocentesis alone can incite synovial inflammation. Additionally, the same joint was not treated during the second study period. While the reason for treatment of another joint was to further safety assuming differential responses of joints to inflammation, repeat administration of an allogeneic product within the same joint may have elicited a greater local immune response. Horses were not free of lameness for this study and treatment was randomly assigned to the limb, meaning the limb treated may or may not have been the limb responsible for the primary lameness. Joints were deemed normal by radiographic screening which only captures bone changes in response to articular pathology. Subtle changes to the gait in the treated limbs may have gone unnoticed due to the initial magnitude or worsening of the baseline lameness present due to changes in husbandry (stall vs. turnout). Though objective lameness data was obtained in this study, another limitation is the lack of objective data for heat and inflammation assessment of treated joints. Infrared thermography (IRT) could be integrated into future studies to detect subclinical inflammatory responses, as well as objectively correlate temperature changes with clinical observations. Cytokine analysis of the alloPRP product was not performed. This was a safety study focused on understanding the systemic and local effects following intra-articular alloPRP administration without intent to evaluate product composition and/or clinical efficacy. Detailed investigation of the proteome of the product along with clinical efficacy is warranted for future studies.

## 5 Conclusion

In this study, we evaluated the effects of pooled allogeneic freeze-dried equine PRP in normal joints compared to saline control. Both treatment and control groups displayed a mild, self-limiting inflammatory response; however, no increase in lameness was observed. The formulation proposed in this project demonstrated to be safe for use in equine joints. Further investigation is necessary for evaluation of clinical efficacy of this product.

## Data Availability

The raw data supporting the conclusions of this article will be made available by the authors, without undue reservation.
